# Tuberculosis incidence in Brazil: time series analysis between 2001 and 2021 and projection until 2030

**DOI:** 10.1590/1980-549720240027

**Published:** 2024-06-14

**Authors:** Marcus Tolentino Silva, Taís Freire Galvão

**Affiliations:** I Universidade de Brasília, School of Health Sciences, Public Health Department – Brasília (DF), Brazil.; II Universidade Estadual de Campinas, School of Pharmaceutical Sciences – Campinas (SP), Brazil.

**Keywords:** Tuberculosis, Incidence, Epidemiological models, Interrupted time series analysis

## Abstract

**Objective:**

To assess the incidence of tuberculosis in Brazil between 2001 and 2022 and estimate the monthly incidence forecast until 2030.

**Methods:**

This is a time-series study based on monthly tuberculosis records from the Notifiable Diseases Information System and official projections of the Brazilian population. The monthly incidence of tuberculosis from 2001 to 2022 was evaluated using segmented linear regression to identify trend breaks. Seasonal autoregressive integrated moving average (Sarima) was used to predict the monthly incidence from 2023 to 2030, deadline for achieving the sustainable development goals (SDGs).

**Results:**

There was a decrease in incidence between January/2001 and December/2014 (4.60 to 3.19 cases-month/100,000 inhabitants; β=-0.005; p<0.001), followed by an increase between January/2015 and March /2020 (β=0.013; p<0.001). There was a sharp drop in cases in April/2020, with the onset of the pandemic, and acceleration of the increase in cases since then (β=0.025; p<0.001). A projection of 124,245 cases in 2030 was made, with an estimated incidence of 4.64 cases-month/100,000 inhabitants, levels similar to those in the 2000s. The Sarima model proved to be robust, with error of 4.1% when removing the pandemic period.

**Conclusion:**

The decreasing trend in tuberculosis cases was reversed from 2015 onwards, a period of economic crisis, and was also impacted by the pandemic when there was a reduction in records. The Sarima model can be a useful forecasting tool for epidemiological surveillance. Greater investments in prevention and control need to be made to reduce the occurrence of tuberculosis, in line with the SDGs.

## INTRODUCTION

Tuberculosis is a global health concern, and its monthly incidence is a major indicator for epidemiological surveillance systems. Such data help to understand the disease trends and to develop control strategies, considering factors such as seasonality, socioeconomic conditions, vaccination coverage, access to health services, quality in diagnosis and notification^1^.

In Brazil, the epidemiological surveillance of tuberculosis is carried out by the National Tuberculosis Control Program (NTCP), from the Unified Health System (SUS), which monitors the disease situation and trend^2^. NTCP collects and shows data, which can be improved with robust and innovative statistical methods, as well as employed in the monitoring of the global sustainable development goals (SDGs), from the United Nations (UN). The target 3.3 of the SDGs, specifically, aims at ending the tuberculosis pandemic by 2030^3^. In this sense, studying the trend based on official data and forecasting new cases throughout time can provide relevant evidence for the guidance of actions to reach these objectives.

Among the time series analysis strategies, the segmented regression for structural break detection and the seasonal autoregressive integrated moving average (Sarima) stand out^4^. Sarima, which is an extension of Arima (autoregressive integrated moving average), is used to model time series with seasonal patterns, trends and other non-seasonal patterns. This model has been employed in studies to forecast cases of tuberculosis in different countries^5-9^, with potential to be used in Brazilian surveillance systems to improve the quality and reliability of the information.

This study aims at assessing the incidence of tuberculosis in Brazil between 2001 and 2022 and to estimate the forecasted monthly incidence until 2030.

## METHODS

This is a time series analysis based on the incidence records of tuberculosis in Brazil from 2001 to 2022, and forecast of new cases until 2030. This study followed the recommendations from “The reporting of studies conducted using Observational Routinely-collected health Data (Record) statement”^10^.

New cases of tuberculosis registered in the Notifiable Diseases Information System (Sinan) in Brazil, between January, 2001, and December, 2022, were eligible. Sinan is the official system for the notification and investigation of diseases and health issues that integrate the national list of reportable diseases^11^. New cases of tuberculosis consider individuals whose tuberculosis diagnosis was confirmed by sputum smear microscopy, culture or clinical diagnosis for situations in which the physician, supported by clinical and epidemiological data and complementary examinations, established the tuberculosis diagnosis in individuals who had never taken specific drugs against the disease^2^. Therefore, it included cases of pulmonary and extrapulmonary tuberculosis, also diagnosed by rapid molecular test.

The Brazilian population estimates for the period between 2001 and 2030 was obtained from the annual population projections in the Brazilian Institute of Geography and Statistics (IBGE)^12^. The population projection and the monthly cases of tuberculosis of each year were inserted in a spreadsheet to compose the database of the study, containing the following variables: year, month, number of cases and size of population. The monthly tuberculosis incidence was calculated subsequently, and expressed in cases-month/100 thousand inhabitants, providing a standardized metric to assess the incidence of the disease throughout time. There was no data imputation.

We performed an exploratory analysis to visualize the data series and to identify patterns, trends and seasonality, analyzed in segmented linear regression to detect inflection points in trendlines^13^. The dataset was then divided in two parts^14^: training dataset (from January 2001 to June 2018, 80% of the cases) and test dataset for the model validation (from July, 2018, to December, 2022, 20% of the cases). In the training dataset, to determine if the series was stationary, the Phillips-Perron and Dickey Fuller tests were used for unit roots. The selection of parameters for Sarima was oriented by the inspection of autocorrelation function (ACF) and partial autocorrelation function (PACF), besides the likelihood and the Akaike information criterion (AIC) and the Bayesian information criterion (BIC)^15^. The lags (peaks) of ACF and PACF, which provide information about the temporal structure of the data, were limits to test the Sarima parameters. Considering these limits, the parameters of the Sarima models identified in the literature for tuberculosis were also tested^16-20^. Based on the selected Sarima model, we forecasted the incidence of tuberculosis for the test period (from July, 2018, to December, 2022). The accuracy of the model was assessed by the mean squared error (MSE), which quantifies the difference between forecasts and the observed values, and by the mean absolute percentage error (Mape), which expresses error as percentage^15^, with sensitivity analysis excluding the period from April 2020 to May 2021, in order to remove the COVID-19 pandemic effect in this accuracy. Then, with the complete dataset, the forecasted incidence of tuberculosis between January, 2023, to December, 2030 was calculated, with 95% confidence intervals (95%CI). Finally, the residual generated by the selected model was assessed using graphic techniques and statistics, including the Ljung-Box Q test for white noise^17^. All analysis routines were executed in Stata 14.2^21^.

The research was based on open database records, available at http://tabnet.datasus.gov.br/cgi/tabcgi.exe?sinannet/cnv/tubercbr.def, not requiring an appreciation from a Research Ethics Committee.

## RESULTS

We included 1,956,616 cases of tuberculosis between 2001 and 2022, whose monthly incidence ranged from 4.92 cases-month/100 thousand inhabitants in May, 2002, to 3.01 cases-month/100 thousand inhabitants in February and March, 2015 (Table 1). After the beginning of the COVID-19 pandemic, lower monthly incidence was registered (2.94 cases-month/100 thousand inhabitants in May, 2020). We observed that 2002 had the highest monthly mean of cases (4.34 cases-month/100 thousand inhabitants), and the highest annual incidence (52.09 cases-year/100 thousand inhabitants).

**Table 1:** Monthly incidence of tuberculosis cases in Brazil per 100 thousand inhabitants.

Year	Jan.	Feb.	Mar.	Apr.	May	Jun.	Jul.	Aug.	Sep.	Oct.	Nov.	Dec.
2001	4.60	3.72	4.60	4.15	4.35	3.87	3.97	4.59	3.81	4.28	3.91	3.77
2002	4.49	4.12	4.47	4.92	4.37	3.77	4.34	4.67	4.29	4.60	4.22	3.82
2003	4.49	4.42	4.14	4.42	4.44	3.92	4.40	4.20	4.50	4.72	4.22	4.04
2004	4.14	3.69	4.69	4.42	4.24	4.00	4.28	4.50	4.32	4.38	4.24	3.93
2005	4.01	3.64	4.56	4.30	4.33	4.20	3.93	4.53	4.15	3.88	4.10	4.07
2006	3.94	3.57	4.34	3.63	4.05	3.73	3.79	4.12	3.63	3.74	3.56	3.28
2007	3.88	3.27	4.28	3.84	3.89	3.48	3.79	3.99	3.54	3.94	3.53	3.22
2008	3.84	3.47	3.80	3.97	3.60	3.62	4.01	4.07	3.98	3.95	3.55	3.43
2009	3.68	3.33	4.21	3.88	3.70	3.46	3.85	3.81	3.82	3.76	3.63	3.47
2010	3.54	3.28	4.25	3.61	3.57	3.39	3.65	3.78	3.67	3.69	3.60	3.67
2011	3.60	3.68	3.79	3.83	3.89	3.52	3.59	4.05	3.74	3.54	3.74	3.51
2012	3.70	3.40	3.96	3.48	3.81	3.41	3.64	4.02	3.38	3.82	3.48	3.16
2013	3.73	3.09	3.53	3.83	3.48	3.46	3.67	3.95	3.67	3.87	3.45	3.15
2014	3.78	3.47	3.33	3.58	3.54	3.12	3.71	3.51	3.70	3.68	3.41	3.19
2015	3.48	3.01	3.83	3.37	3.37	3.38	3.68	3.65	3.52	3.59	3.56	3.35
2016	3.45	3.30	3.91	3.57	3.55	3.65	3.39	3.74	3.41	3.20	3.42	3.24
2017	3.65	3.29	4.19	3.27	3.91	3.57	3.49	3.91	3.59	3.77	3.60	3.38
2018	3.82	3.27	3.80	3.90	3.78	3.71	3.89	4.27	3.67	4.17	3.69	3.32
2019	3.97	3.69	3.63	3.93	3.98	3.50	4.00	3.91	3.88	4.09	3.63	3.45
2020	4.12	3.51	4.07	2.94	2.67	3.07	3.36	3.34	3.56	3.55	3.38	3.17
2021	3.26	3.19	3.56	3.28	3.29	3.46	3.66	3.93	3.94	3.74	3.89	3.81
2022	3.74	3.85	4.45	3.83	4.16	3.79	4.03	4.40	4.02	3.91	3.65	3.60

**Figure 1:**
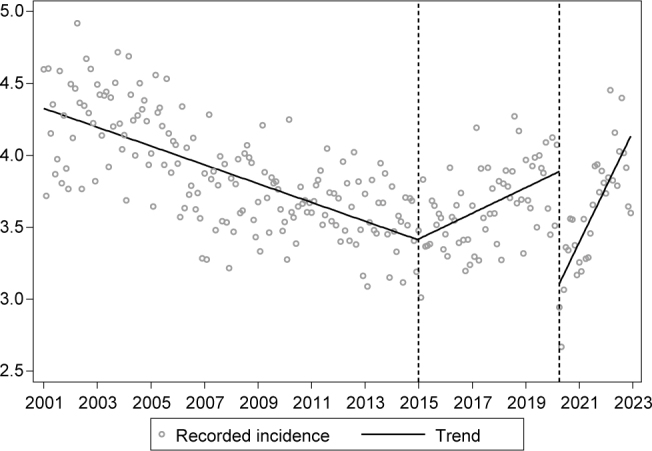
Monthly incidence of tuberculosis per 100 thousand inhabitants in Brazil between 2001 and 2022.

The monthly incidence between January, 2001, and December, 2014, presented a decreasing trend (β=-0.005; p<0.001) (Figure 1). Between January, 2015, and March, 2020, there was an increasing trend (β=0.013; p<0.001). There was an abrupt drop in April, 2020 (β=-0.781; p<0.001), with the onset of the pandemic. In the subsequent months, until December, 2022, there was acceleration in the number of cases (β=0.025; p<0.001).

**Figure 2:**
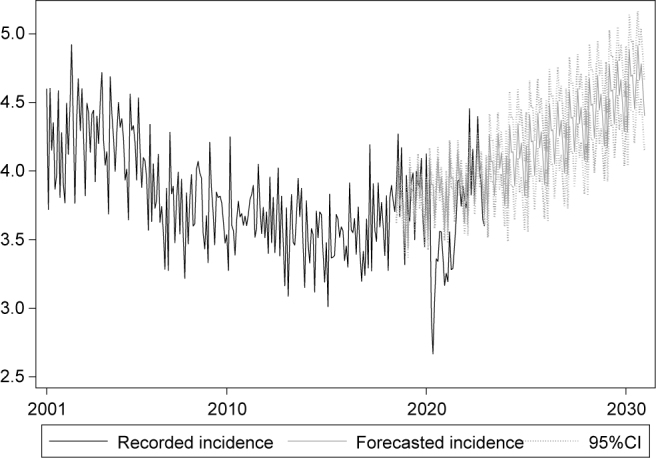
Monthly incidence of tuberculosis per 100 thousand inhabitants in Brazil between 2001 and 2030.

Maintaining the observed trends, based on the Sarima model, the incidence between 2023 and 2023 shows itself to be increasing (Figure 2).

**Figure 3:**
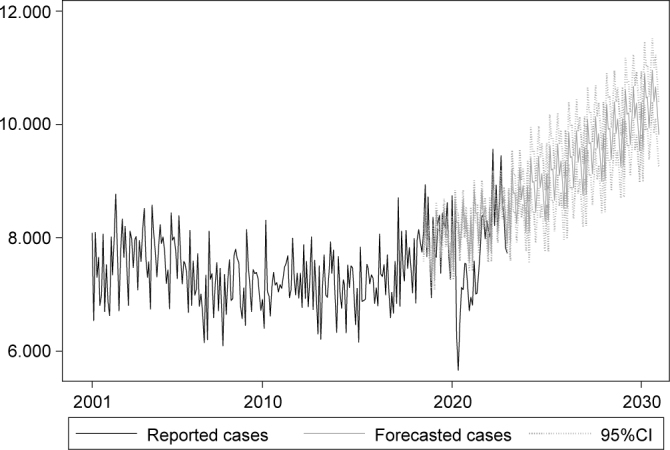
Number of monthly cases of tuberculosis in Brazil between 2001 and 2030.

At the end of the series, the incidence was estimated in 55.68 cases/100 thousand inhabitants a year, the highest in the period, estimating 4.40 cases-month/100 thousand inhabitants (95%CI 4.15-4.65), in December 2030, with higher monthly incidence in August of that year (Table 2). In absolute terms, the estimate is that 124,245 new cases will be recorded in 2030, with more than 10 thousand cases/month (Figure 3), with estimated monthly incidence of 4.64 cases-month/100 thousand inhabitants in 2030.

**Table 2:** Monthly estimated incidence of tuberculosis per 100 thousand inhabitants in Brazil between 2023 and 2030.

Month	2023	2024	2025	2026	2027	2028	2029	2030
Jan.	4.04	4.11	4.18	4.26	4.35	4.45	4.55	4.65
Feb.	3.66	3.73	3.81	3.89	3.98	4.07	4.17	4.28
Mar.	4.27	4.33	4.41	4.49	4.58	4.68	4.78	4.89
Apr.	4.07	4.14	4.21	4.29	4.38	4.48	4.58	4.69
May	4.07	4.14	4.22	4.30	4.39	4.49	4.59	4.70
Jun.	3.82	3.89	3.97	4.05	4.14	4.24	4.34	4.45
Jul.	4.03	4.10	4.18	4.26	4.35	4.45	4.55	4.66
Aug.	4.27	4.34	4.42	4.51	4.60	4.70	4.80	4.91
Sep.	4.02	4.09	4.17	4.25	4.35	4.45	4.55	4.66
Oct.	4.13	4.20	4.28	4.37	4.46	4.56	4.67	4.78
Nov.	3.95	4.02	4.10	4.19	4.28	4.38	4.49	4.60
Dec.	3.75	3.82	3.90	3.99	4.08	4.18	4.29	4.40
n	103,847	106,255	108,831	111,575	114,488	117,571	120,824	124,245
A/I	48.08	48.92	49.85	50.85	51.94	53.11	54.35	55.68

n: total estimated cases in the year; A/I: estimated annual incidence.

The supplementary material reports the selection of parameters for Sarima. The (2,1,1)x(1,1,1)^12^ model demonstrated the best performance in the training base. When forecasting the period of the test base, the model showed an 8.0% error considering the COVID-19 pandemic. When removing the pandemic period, Mape decreased to 4.5%. Residual analysis confirmed the model robustness.

## DISCUSSION

After a decade and a half of reduction in the incidence of tuberculosis, from 2015 onwards, cases began to increase in Brazil, until the beginning of the COVID-19 pandemic, when there was a drop; since then, a new increase in incidence is observed until 2022. If this pattern is maintained, the estimate is that, until 2030, the incidence will remain growing, resuming the levels of the 2000s, the beginning of the historical series, which undermines the national commitment towards the tuberculosis targets included in the SDGs.

Present results are limited by different factors, which can affect their validity and generalization. The data come from Sinan reporting, which can range in quality in the different stages of identification, investigation, and laboratory confirmation of tuberculosis according to the technical and operational conditions of the regional epidemiological surveillance system^22^. The study did not distinguish the several clinical forms of the disease, each one with their different implications in the transmission and understanding of the condition. These factors, which are beyond the scope of this research, can affect the accuracy of the model forecasts. The COVID-19 pandemic may have affected the expected reporting pattern between 2020 and 2021^23^. To mitigate this limitation, the total results were compared with an analysis that excluded this period in the assessment of model accuracy. The adopted model did not consider external factors that can influence the incidence of tuberculosis, such as socioeconomic conditions and access to health services; also, when using aggregated data at a national level, the study may have masked regional heterogeneities that are known about the occurrence of tuberculosis in Brazil^24-26^. The updated population projections based on the Demographic Census of 2022 were not available and may have underestimated the incidence of tuberculosis, and thus affected the calculation of the future trends. The employment of the advanced technique to forecast time series, on the other hand, allowed to capture seasonal and non-seasonal patterns of the data and to provide forecasts until 2030, which can be valuable for the monitoring of the SDGs, with low error for the adopted model.

This study investigated the trend of tuberculosis cases in Brazil and observed increasing incidence coinciding with austerity policies at the end of the historical series. Previous national studies corroborate the reduction and growth trends observed in our analyses, also investigating regional factors and specific groups^24-26^.

The study identified inflection points in the trends of tuberculosis cases, which may be related to historical events or structural changes in the disease dynamics. Between 2001 and 2015, there was a consistent reduction in the incidence of tuberculosis in Brazil. This reduction coincided with a phase of economic growth and reduction of inequalities in the country, marked by investments in social programs, increased coverage in primary health care and infrastructure of SUS^27^. After 2015, there was a persistent increase in cases of tuberculosis, which may be attributed to several factors, including the economic slowdown and cuts to social programs, with general decrease in social welfare^28^. If the observed trends continue without any intervention, the projections indicate that disease control in 2030 will regress to the worst levels observed in the 2000s. This throwback would be a major setback in the efforts to eradicate tuberculosis, with serious implications for public health. To reach the SDGs and significantly decrease the incidence of tuberculosis, it is strategic to change the current course of the disease, which will require a multifaceted approach that includes robust medical, political, economic, and social interventions^3^. Investment in prevention, early diagnosis, adequate treatment and support to vulnerable communities will be essential. In this sense, strengthening SUS is fundamental to ensure public policies are aligned with the long-term goals of control and eradication of tuberculosis.

The Sarima model we used has broad application in several contexts, and can be combined with other techniques to predict the incidence of tuberculosis. An analysis in Mainland China about the temporal trends of tuberculosis morbidity used the hybrid Sarima-NARNNX model (nonlinear autoregressive neural network with exogenous input), projecting an annual reduction of 3.002% in the incidence of the disease until 2025^5^. Another study used a variety of methods, including segmented regression, the Sarima model, geographical clustering and multivariate time series, observing a mean annual reduction of 3.3% in the incidence of tuberculosis between 2004 and 2017, with seasonal peaks and geographical clusters in Xinjiang and Tibet^6^. Na analysis of 331,594 cases reported between 2009 and 2018 in the province of Zhejiang, China, revealed a decreasing trend in incidence, from 75.38/100,000 to 52.25/100,000, identifying seasonal peaks in March and April, and the Sarima model showed specific epidemiological characteristics and sex ratio of 2:1 (male x female)^9^. In the United States of America, a study used the Sarima model to investigate a 20% reduction in cases of tuberculosis during the COVID-19 pandemic, comparing the mean of the years 2016 to 2019, using data regarding drug sales^7^. In South Africa, the superiority of Sarima-NNAR (Neural Network Autoregression) in comparison to Sarima to forecast the incidence of tuberculosis stood out, emphasizing the need for interventions during festivals^8^. This technique has proven to be relevant in the mid and long-term monitoring of tuberculosis, providing valuable evidence for the surveillance and prevention of the disease.

In conclusion, if the current trends are maintained, the incidence of tuberculosis will continue to increase and, in 2030, return to the levels registered in the 2000s. The Sarima model has proven to be useful to estimate the incidence of tuberculosis in Brazil. Integrated and intersectoral actions to reduce social inequalities and improve access to health services for the most vulnerable populations should be prioritized. Detailed analyses per region, state or city, and per specific population groups, such as indigenous people, quilombola communities and people deprived of liberty can bring specific evidence in future studies. The exploration of other forecasting techniques that include external or non-linear factors in the modeling of tuberculosis incidence may also be useful, allied with simulations to assess the potential impact of different epidemiological scenarios or sanitary interventions in the evolution of the disease.
