# Supplementary material for: Tuberculosis incidence in Brazil: time series analysis between 2001 and 2021 and projection until 2030
Source: Rev Bras Epidemiol. 2024 Jun 14;27:e240027. doi: 10.1590/1980-549720240027 (PMC11182439; doi:10.1590/1980-549720240027)
Supplement: Supplementary file 1 [file 1980-5497-rbepid-27-e240027-Material-suplementar.docx]

Incidência de tuberculose no Brasil: análise de série temporal e tendência futura

Material suplementar

No banco treinamento, os testes Phillips-Perron e Dickey-Fuller confirmaram que a série não é estacionária (p<0,001). O ACF indicou uma defasagem significativa para a ordem de médias móveis (Figura Suplementar 1-A). O PACF apontou três defasagens significativas para o componente autorregressivo (Figura Suplementar 1-B). Ao considerar a sazonalidade, tanto o ACF (Figura Suplementar 1-C) quanto o PACF (Figura Suplementar 1-D) indicaram uma defasagem significativa.

A investigação de sete modelos SARIMA para prever casos de tuberculose indicou que o 6, especificado como (2,1,1)x(1,1,1)12 demonstrou o melhor desempenho, com o maior valor de verossimilhança e os menores valores de AIC e BIC (Tabela Suplementar 1).

Após aplicar o modelo SARIMA selecionado no banco teste (Tabela Suplementar 2), foi observado erro absoluto (MSE) de 0,14 e erro percentual (MAPE) de 8,0%. Ao remover o período da pandemia, o MSE reduziu para 0,03 e o MAPE para 4,10%.

A avaliação dos resíduos mostrou a robustez do modelo. O gráfico do resíduo padronizado em série temporal destaca a influência da pandemia na estimativa (Figura Suplementar 2-A). O gráfico de densidade dos resíduos aproxima-se da normalidade (p = 0,0681) (Figura Suplementar 2-B). O valor p do teste de ruído branco com Ljung-Box Q foi 0,414, alterando-se para 0,989 ao remover o período da pandemia. O gráfico que avalia a estabilidade das estimativas, mostra que as raízes autorregressivas, representadas por pontos dentro do círculo do gráfico, são estáveis e inversíveis. A proximidade das raízes com a borda do círculo e o padrão de distribuição das raízes enfatizam a sensibilidade e sazonalidade do modelo.

Figura Suplementar 1. Gráficos das funções de autocorrelação (ACF) e autocorrelação parcial (PACF).

| A | B |
| --- | --- |
|  |  |
| C | D |
|  |  |

Notas: A: ACF do componente médias móveis do componente não-sazonal. B: PACF do componente autorregressivo do componente não-sazonal. C: ACF do componente médias móveis do componente sazonal. D: PACF do componente autorregressivo do componente sazonal.

Tabela Suplementar 1. Seleção do modelo SARIMA para predição dos casos de tuberculose.

| Parâmetro | Modelo 1 | Modelo 2 | Modelo 3 | Modelo 4 | Modelo 5 | Modelo 6 | Modelo 7 |
| --- | --- | --- | --- | --- | --- | --- | --- |
| p | 0 | 1 | 1 | 1 | 2 | 2 | 3 |
| d | 1 | 0 | 0 | 1 | 0 | 1 | 1 |
| q | 1 | 0 | 0 | 1 | 0 | 1 | 1 |
| P | 0 | 0 | 1 | 1 | 0 | 1 | 1 |
| D | 1 | 1 | 1 | 1 | 1 | 1 | 1 |
| Q | 1 | 1 | 1 | 1 | 1 | 1 | 1 |
| s | 12 | 12 | 12 | 12 | 12 | 12 | 12 |
| V | 24,99 | 4,56 | 12,71 | 30,60 | 13,10 | 31,87* | 30,01 |
| AIC | -41,98 | -1,11 | -17,43 | -49,20 | -16,21 | -51,75* | -44,02 |
| BIC | -28,85 | 12,04 | -4,27 | -29,50 | 0,23 | -32,05* | -17,76 |

Notas: p: Ordem do componente autoregressivo. Representa o número de defasagens (lags) da série temporal utilizadas como preditores. d: Ordem da diferenciação. Indica o número de vezes que a série temporal foi diferenciada para alcançar estacionariedade. q: Ordem do componente de médias móveis. Representa o número de defasagens dos erros utilizadas como preditores. P: Ordem do componente autoregressivo sazonal. Indica o número de defasagens sazonais da série temporal utilizadas como preditores. D: Ordem da diferenciação sazonal. Reflete o número de vezes que a série temporal foi diferenciada em defasagens sazonais para alcançar estacionariedade sazonal. Q: Ordem do componente de médias móveis sazonal. Representa o número de defasagens sazonais dos erros utilizadas como preditores. s: Período de sazonalidade. Refere-se ao número de observações em cada ciclo sazonal. V: Verossimilhança. Modelos com valores maiores são preferíveis. AIC: Critério de Informação de Akaike. Modelos com valores menores são preferíveis. BIC: Critério de Informação Bayesiano. Modelos com valores menores são preferíveis. *: Aponta os melhores resultados encontrados.

Tabela Suplementar 2. Erro absoluto e erro percentual das previsões mensais do modelo SARIMA (2,1,1)x(1,1,1)_12_ com dados reais de incidência de tuberculose para 100.000 habitantes.

| Ano | M | R | Est | Δ abs | Δ % |  | Ano | Mês | R | Est | Δ abs | Δ % |
| --- | --- | --- | --- | --- | --- | --- | --- | --- | --- | --- | --- | --- |
| 2018 | Jul | 3,89 | 3,70 | 0,19 | 4,9 |  | 2020 | Out | 3,55 | 3,96 | -0,40 | 11,3 |
| 2018 | Ago | 4,27 | 4,00 | 0,28 | 6,5 |  | 2020 | Nov | 3,38 | 3,77 | -0,39 | 11,6 |
| 2018 | Set | 3,67 | 3,75 | -0,08 | 2,2 |  | 2020 | Dez | 3,17 | 3,57 | -0,40 | 12,6 |
| 2018 | Out | 4,17 | 3,86 | 0,31 | 7,4 |  | 2021 | Jan | 3,26 | 3,93 | -0,67 | 20,6 |
| 2018 | Nov | 3,69 | 3,67 | 0,02 | 0,5 |  | 2021 | Fev | 3,19 | 3,55 | -0,36 | 11,1 |
| 2018 | Dez | 3,32 | 3,47 | -0,15 | 4,6 |  | 2021 | Mar | 3,56 | 4,15 | -0,59 | 16,6 |
| 2019 | Jan | 3,97 | 3,84 | 0,13 | 3,2 |  | 2021 | Abr | 3,28 | 3,95 | -0,67 | 20,3 |
| 2019 | Fev | 3,69 | 3,44 | 0,25 | 6,8 |  | 2021 | Mai | 3,29 | 3,95 | -0,66 | 20,1 |
| 2019 | Mar | 3,63 | 4,02 | -0,39 | 10,7 |  | 2021 | Jun | 3,46 | 3,70 | -0,24 | 7,1 |
| 2019 | Abr | 3,93 | 3,87 | 0,06 | 1,4 |  | 2021 | Jul | 3,66 | 3,91 | -0,25 | 6,8 |
| 2019 | Mai | 3,98 | 3,85 | 0,13 | 3,3 |  | 2021 | Ago | 3,93 | 4,15 | -0,23 | 5,8 |
| 2019 | Jun | 3,50 | 3,63 | -0,13 | 3,7 |  | 2021 | Set | 3,94 | 3,90 | 0,04 | 1,1 |
| 2019 | Jul | 4,00 | 3,80 | 0,20 | 5,0 |  | 2021 | Out | 3,74 | 4,01 | -0,26 | 7,1 |
| 2019 | Ago | 3,91 | 4,05 | -0,14 | 3,6 |  | 2021 | Nov | 3,89 | 3,82 | 0,07 | 1,8 |
| 2019 | Set | 3,88 | 3,80 | 0,08 | 2,1 |  | 2021 | Dez | 3,81 | 3,62 | 0,19 | 5,0 |
| 2019 | Out | 4,09 | 3,91 | 0,18 | 4,5 |  | 2022 | Jan | 3,74 | 3,98 | -0,25 | 6,6 |
| 2019 | Nov | 3,63 | 3,72 | -0,09 | 2,5 |  | 2022 | Fev | 3,85 | 3,60 | 0,24 | 6,3 |
| 2019 | Dez | 3,45 | 3,52 | -0,07 | 2,0 |  | 2022 | Mar | 4,45 | 4,20 | 0,25 | 5,6 |
| 2020 | Jan | 4,12 | 3,88 | 0,24 | 5,9 |  | 2022 | Abr | 3,83 | 4,01 | -0,18 | 4,7 |
| 2020 | Fev | 3,51 | 3,50 | 0,01 | 0,4 |  | 2022 | Mai | 4,16 | 4,01 | 0,15 | 3,6 |
| 2020 | Mar | 4,07 | 4,10 | -0,02 | 0,6 |  | 2022 | Jun | 3,79 | 3,76 | 0,03 | 0,8 |
| 2020 | Abr | 2,94 | 3,90 | -0,96 | 32,6 |  | 2022 | Jul | 4,03 | 3,96 | 0,06 | 1,6 |
| 2020 | Mai | 2,67 | 3,90 | -1,23 | 46,2 |  | 2022 | Ago | 4,40 | 4,21 | 0,19 | 4,3 |
| 2020 | Jun | 3,07 | 3,65 | -0,59 | 19,2 |  | 2022 | Set | 4,02 | 3,95 | 0,06 | 1,5 |
| 2020 | Jul | 3,36 | 3,85 | -0,49 | 14,7 |  | 2022 | Out | 3,91 | 4,07 | -0,15 | 3,9 |
| 2020 | Ago | 3,34 | 4,10 | -0,76 | 22,7 |  | 2022 | Nov | 3,65 | 3,88 | -0,23 | 6,4 |
| 2020 | Set | 3,56 | 3,84 | -0,28 | 8,0 |  | 2022 | Dez | 3,60 | 3,68 | -0,08 | 2,3 |

Notas: M: meses. R: valor real observado. Est: valor estimado no modelo. Δ abs: diferença absoluta entre os valores reais e estimados. Δ %: erro percentual entre a previsão e os dados reais.

Figura Suplementar 2. Avaliação dos resíduos e estabilidade do modelo SARIMA (2,1,1)x(1,1,1)_12_.

| A |
| --- |
|  |
| B |
|  |
| C |
|  |

Notas: A: comportamento dos resíduos ao longo da série temporal. B: investigação da normalidade dos resíduos. C: Investigação da estabilidade das raízes do componente autorregressivo (AR) e das médias móveis (MA), valores estimados no eixo x e valores reais no eixo y.
